# Evaluation of mechanistic and statistical methods in forecasting influenza-like illness

**DOI:** 10.1098/rsif.2018.0174

**Published:** 2018-07-25

**Authors:** Sasikiran Kandula, Teresa Yamana, Sen Pei, Wan Yang, Haruka Morita, Jeffrey Shaman

**Affiliations:** Department of Environmental Health Sciences, Columbia University, New York, NY, USA

**Keywords:** influenza, forecasts, mechanistic models, meta-ensemble, nowcast

## Abstract

A variety of mechanistic and statistical methods to forecast seasonal influenza have been proposed and are in use; however, the effects of various data issues and design choices (statistical versus mechanistic methods, for example) on the accuracy of these approaches have not been thoroughly assessed. Here, we compare the accuracy of three forecasting approaches—a mechanistic method, a weighted average of two statistical methods and a super-ensemble of eight statistical and mechanistic models—in predicting seven outbreak characteristics of seasonal influenza during the 2016–2017 season at the national and 10 regional levels in the USA. For each of these approaches, we report the effects of real time under- and over-reporting in surveillance systems, use of non-surveillance proxies of influenza activity and manual override of model predictions on forecast quality. Our results suggest that a meta-ensemble of statistical and mechanistic methods has better overall accuracy than the individual methods. Supplementing surveillance data with proxy estimates generally improves the quality of forecasts and transient reporting errors degrade the performance of all three approaches considerably. The improvement in quality from ad hoc and post-forecast changes suggests that domain experts continue to possess information that is not being sufficiently captured by current forecasting approaches.

## 1. Introduction

In the USA, an estimated 9–35 million influenza infections occur annually, with 140 000–710 000 resulting hospitalizations and 12 000–56 000 deaths [1,2]. Public health agencies such as the Centers for Disease Control and Prevention (CDC) have built surveillance systems to collect and disseminate influenza outbreak information in near real time [3,4]. While these systems provide essential situational awareness of influenza activity, tools that accurately and reliably predict outbreak characteristics, such as peak timing and magnitude, can aid decision makers in implementing control and mitigation strategies.

Several groups have proposed a variety of mechanistic and statistical methods to forecast seasonal and pandemic influenza [5,6]. Broadly, statistical methods model outbreaks as time series and do not directly account for disease transmission dynamics [7–12], whereas mechanistic methods model disease states either at the population level [13–16] or at the more computationally expensive individual level [17–19]. More recently, there has been evidence that collective human judgement has considerable predictive power, and that for some of the outcomes, it can match or exceed most statistical and mechanistic methods [20,21].

Additionally, to supplement surveillance data with more up-to-date information, methods to nowcast (i.e. provide estimates of incidence during more recent weeks for which surveillance data are not yet available) and forecast influenza using online search trends [22–24], twitter feeds [25–29], access logs of influenza-related webpages at Wikipedia [30,31] and CDC [32], online news and informal reports [33,34], electronic health records [35] and combinations of these data sources [32,36] have also been proposed. Given this abundance of nowcasting and forecasting methods, approaches for combining or weighting these different methods have been explored. In particular, given that the advantage of statistical models over mechanistic models during a season tends to be inversely related to the deviation of the season's influenza activity from a typical season, an ensemble that combines a variety of diverse forecast methods including both statistical and mechanistic models could reduce forecast uncertainty and outperform either type of method. Findings from numerical weather prediction strongly suggest that ensembles of disparate models would at least match the best performing ensemble member [37,38]. Similarly, recent studies on the application of ensemble approaches to infectious disease forecasting have reported promising improvements [39–41].

Beginning with the 2013–2014 season, CDC's Influenza Division has been coordinating with influenza modelling groups to assemble real-time weekly influenza forecasts at the US National and Health and Human Services (HHS) regional levels [42]. This collaborative, the Epidemic Prediction Initiative's FluSight [43], has identified forecasting targets that would be useful to decision makers, defined templates for sharing forecasts across teams and established robust evaluation metrics.

Here, we describe three methods—a mechanistic model-inference method, a weighted average of two statistical methods and a super-ensemble of eight statistical and mechanistic models—that we used during the 2016–2017 influenza season to generate point and probabilistic forecasts in real time for the FluSight competition. We compare and report on the relative accuracy of the three methods in predicting seven targets of interest, as evaluated using two measures—a logarithmic scoring of the probabilistic forecasts and the mean absolute error of the point forecasts.

In addition to comparing the three forecast methods above, we quantify the effects of nowcasts, post-processing and data reporting issues on forecast accuracy. First, we measure the improvement in forecast accuracy resulting from the use of nowcasts as supplements to near real-time ILI surveillance data. Second, we report the effect of post-processing and of ad hoc modifications based on expert judgement, on the forecast quality. Lastly, as the surveillance data are revised over multiple weeks in response to updated reports from participating clinics, forecasts made in real time are based on transient estimates of ILI. We report the impact of these initial under- or over-estimates of ILI on the accuracy of forecasts produced with each method.

## 2. Material and methods

### 2.1. Overview

For each of the 10 HHS regions and the US national level during each week of the influenza season, we generated forecasts using three different approaches, namely: (i) DYN: a model-inference ensemble forecast using a compartmental model coupled with state space estimation and dynamic error growth correction; (ii) STAT: a weighted average of two statistical forecasting methods based on weighted combinations of historical outbreak trajectories; (iii) SE: a super-ensemble of six model-inference forecasting variants and the two statistical forecasting methods in (ii).

Additionally, as there is generally a week's lag between the end of a week and the public release of the week's ILI through CDC's FluView interface, we estimated ILI activity for the lagged week using search query data from Google Extended Health Trends (GET) API and other online sources such as Twitter and Wikipedia access logs [32,44,45]. We refer to the ILI estimate for this additional week as a nowcast. The forecasts are generated using the time series produced by appending the latest nowcast to the CDC-released ILI estimates.

### 2.2. Nowcasts

To generate weekly nowcasts, we built random forest regression models [46–48] at the national and the HHS regional levels, using weighted ILI [3] reported by the CDC as the response variable and queries whose search patterns are well correlated with ILI as explanatory variables [45]. These correlates were identified from multiple sources including Google Correlate [49], related prior work [50] and an online knowledge base [51].

For each of the correlates identified, we retrieved through the GET API the probability that it was queried during a user's session on Google search engine. The API allows for specification of geographical (country, state, etc.) and temporal (daily, weekly, etc.) granularities and the period of interest. The probabilities are calculated based on a random sample of 10–15% of all searches and are updated daily.

As we are interested in nowcasts at the HHS regional level and GET does not provide separate query fractions at the regional level, we calculated the search frequency for an HHS region as a population-weighted mean of search frequencies from states in the region. We used a ‘weekly’ periodicity to be consistent with the weekly CDC ILI. A logit transformation was applied to the query fractions, as prior work has shown that with logit transformation, the relation between raw query fractions and ILI becomes approximately linear and model performance improves [23].

### 2.3. DYN: model-inference forecasts

The DYN forecast system comprises a mechanistic disease model and a data assimilation method. The mechanistic disease model, describing the propagation of ILI through a population, assumes a susceptible–exposed–infectious–recovered–susceptible (SEIRS) structure per the following equations:

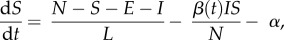


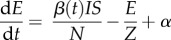


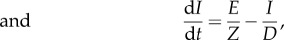
where *S* is the number of susceptible people in the population, *t* is time, *N* is the size of the population, *E* is the number of exposed individuals, *I* is the number of infectious individuals, 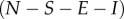 gives the number of recovered individuals, *β*(*t*) is the contact rate at time *t*, *L* is the average duration of immunity, *Z* is the mean latent period, *D* is the mean infectious period and *α* is the rate of travel-related import of infection into the model domain.

The contact rate is calculated as 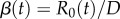, where *R*_0_(*t*) is the basic reproductive number and is modulated by specific humidity, a measure of absolute humidity. Specifically, *R*_0_(*t*) is calculated as follows:

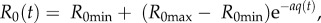
where *R*_0 min_ and *R*_0max_ are the minimum and the maximum daily basic reproductive numbers, respectively, and *q*(*t*) is the time-varying specific humidity. The value of *a* was estimated from the laboratory regression of influenza virus survival upon absolute humidity [52,53]. As in our previous works [13,15,54], instead of observed specific humidity, we used daily specific humidity averaged over 24 years (1979–2002) as this is smoother and yields better forecasts [55]. These local climatological specific humidity data were compiled for 115 cities from the National Land Data Assimilation System (NLDAS) project-2 dataset [56,57]. The climatological specific humidity for an HHS region was an average of the city-level climatological specific humidity of cities in the region. (Specific humidity data are included in the electronic supplementary material.)

Initial parameter values for all simulations were chosen randomly from the following uniform ranges: *R*_0max_ ∼ *U*[1.3, 3.2]; *R*_0 min_ ∼ *U*[0.8, 1.2]; *Z* ∼ *U*[1, 5] days; *D* ∼ *U*[2, 12] days; *L* ∼ *U*[200, 500] days. For all locations, the population size, *N*, was set to 100 000 and the importation rate, *α*, to 0.1 infections per day (1 infection every 10 days).

#### 2.3.1. Ensemble adjustment Kalman filter with error correction

During simulation and prior to generating a forecast, the parameters and variables in the above SEIRS model were iteratively optimized using real-time observations and the ensemble adjustment Kalman filter (EAKF) [58]. The EAKF is a deterministic data assimilation algorithm that is coupled with an ensemble of model simulations. Here, an ensemble of 300 trajectories is randomly initialized, as described above, and integrated per the SEIRS model equations. Upon encountering an observation, the integration is halted; the EAKF uses the first two moments of the ensemble estimate of the observed state variable, the prior, to adjust that ensemble towards the observation, thereby generating a posterior, whose mean and variance are calculated using Bayes' rule. The variance of the distribution is preserved during this update. The posterior is then integrated forward in time to the next observation and the updating process is repeated. In addition, at each update, we also apply an error correction algorithm to counteract the growth of error due to nonlinearity of the disease transmission model [59].

### 2.4. STAT: statistical methods

STAT uses a combination of two statistical forecast methods, Bayesian weighted outbreaks (BWO) and *k*-nearest neighbors (KNN), described below.

#### 2.4.1. Bayesian weighted outbreaks

BWO is a statistical method that uses Bayesian model averaging (BMA) [60–62] to predict the trajectory of ILI during a given season as a weighted average of outbreak trajectories from past seasons. Variations of this method have been used in weather forecasting [38] and in retrospective forecasts of outbreaks of influenza [7,11,40] and dengue [39]. Each previous outbreak, or candidate trajectory, is represented by a normal distribution with mean equal to the observed ILI during the training period (weeks *t* − 5 through *t*) and standard deviation *σ*. We used maximum-likelihood estimation to obtain the candidate trajectory weights *w*_k_ and standard deviation *σ* that best represent the observed ILI during the same training period for the outbreak in progress. These weights were applied to the historical trajectories to predict ILI for weeks *t* + 1 through the end of the influenza season [39]. US National and 10 HHS regional ILI observed during influenza seasons 1997/1998 through 2015–2016 were used as the pool of candidate trajectories for the 2016–2017 influenza season. To account for uncertainty in observed ILI, the BWO process was repeated 100 times, each iteration drawing training data from a Poisson distribution centred on the ILI observations.

#### 2.4.2. *K*-nearest neighbors

Similar to BWO, KNN is an analogue forecast method [11] based on historical outbreak trajectories. The KNN first selects *n* candidate trajectories (i.e. nearest neighbors, *n* = 3 here) based on the distance between the historical trajectories and the most recent observations (i.e. weeks *t* – *k* to *t*; *k* = 4 here). The distance, as in [11], was evaluated based on the sum of squared difference between the observed and historical ILI. Here, the weights for these nearest neighbors at week *t* were computed by minimizing the distance between the weighted-average historical trajectory and the observations. ILI predicted for the following *h* weeks (*h* = 3 here) was then computed as the weighted average (using the optimized weights) of the nearest neighbors in the subsequent *h* weeks. This process was repeated, which iteratively extended the forecast *h* weeks at a time, until ILI for the entire season was predicted. We only used local historical ILI from each location for the optimization and did not match the outbreak time window as in the BWO.

See the electronic supplementary material for the target specific weighting scheme used to combine KNN and BWO.

### 2.5. SE: super-ensemble

Super-ensemble methods allow information from distinct forecast methods to be combined in a statistically rigorous manner to produce a single overall forecast. Super-ensemble forecasts have been shown to be more accurate on average than forecasts produced using a single model or methodology [39–41]. Here, we used the BMA method to produce a weighted-average super-ensemble forecast from eight individual models—six dynamical forecast systems and the two statistical forecasts used in STAT. The dynamical systems used include DYN, as well as five other combinations of dynamical models and filters: SEIRS and SIRS structured mechanistic models, coupled with each of the following data assimilation methods: rank histogram filter (RHF), ensemble Kalman filter (EnKF) and the EAKF used in DYN (see electronic supplementary material, methods).

Model weights were calculated using BMA and are based on the performance of the forecasts produced using these eight methods during previous seasons. The training period used here spans the 2004–2005 through 2015–2016 influenza seasons, excluding the pandemic years of 2008–2009 and 2009–2010. Weights were computed separately for each target and each week. For example, the weights assigned to each of the point estimates of season peak intensity during Morbidity and Mortality Weekly Report (MMWR) week 50 of 2016–2017 were determined by forecasts of season peak intensity at MMWR week 50 during the 2004–2005 through 2015–2016 influenza seasons of each of the eight forecast methods. Training forecasts for the statistical models used a leave-one-out approach, where each season's forecasts were produced using outbreak trajectories for the remaining seasons.

### 2.6. Evaluation

#### 2.6.1. Targets

For US national and each of the 10 HHS regions, forecasts were generated using the three approaches during a large part of the 2016–2017 influenza season—November 2016 to mid-May 2017 (specifically, from MMWR [63] week 44 of 2016 to MMWR week 18 of 2017). To compare the quality of the forecasts, the following targets were used:

— Season onset, defined as the first of three consecutive MMWR weeks for which the observed ILI is greater than the region-specific baseline.^1^ The baselines are published by CDC prior to the start of every season based on influenza activity during the three most recent influenza seasons.
— Season peak intensity, the maximum weekly ILI observed during the season.
— Season peak week, the MMWR week during which the maximum weekly ILI was observed. ILI is traditionally rounded to one decimal point and hence season peak week is not necessarily unique.
— One- to four-week-ahead forecasts, the estimates of ILI one through four weeks beyond the week of forecast initiation. For example, when forecasts are generated using ILI available through MMWR week 50, the one-week-ahead forecast is the ILI estimate for MMWR week 51 and the two-week-ahead forecast is the ILI estimate for MMWR week 52. Here, one-week-ahead forecasts are given by the probabilistic nowcast directly, whereas two- to four-week-ahead forecasts employ the same mechanistic and statistical forecast methods used for seasonal targets.

#### 2.6.2. Probabilistic forecasts

The probabilistic forecast for target *g* at region *r* using ILI available through week *w* is a set of probabilities for the possible outcomes of the target and is denoted by the tuple (region, target and week), henceforth (*r*, *g* and *w*). For season peak week, the possible outcomes are MMWR week 40 through MMWR week 20. For season onset, the possible outcomes are the same as for season peak week plus an additional case to capture the scenario where no onset is forecasted to occur (i.e. ILI does not exceed baseline for more than two consecutive weeks). For the intensity targets, the possible outcomes are intensity intervals of size 0.1% from 0% to 13%, i.e. [0, 0.1), [0.1, 0.2), … , [12.9, 13), and [13, 100]. Electronic supplementary material, figure S1, shows probabilistic forecasts at the national level for all targets. See electronic supplementary material for description on how probabilistic forecasts are calculated in each of the three approaches.

The score of a forecast (*r*, *g*, *w*) is calculated as follows: 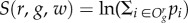, where 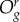 is the set of acceptable outcomes for target *g* at region *r* and *p_i_* is the probability assigned by the forecast to outcome *i*. For season onset and season peak week, the acceptable outcomes are the exact observed week and the two weeks immediately adjacent to it (i.e. ±1 week). For season peak intensity and one- to four-week-ahead forecasts, the acceptable outcomes are the observed intensity interval and the 10 interval bins immediately adjacent to it (i.e. ±0.5%).^2^ For example, if for HHS region 7, the season onset occurred during MMWR week 51, 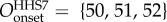 and if the peak ILI was 6.4%, 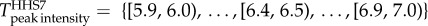.


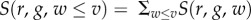
 is the cumulative score for target *g* at region *r* for all weeks up to and including week *v*, and 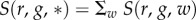 is the cumulative score across all weeks of the season during which forecasts were generated. 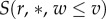, 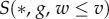, 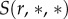 and 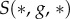 can be defined analogously.

#### 2.6.3. Point forecasts

The point forecast for target *g* at region *r* using ILI available through week *w* is the forecasted value calculated from the mean trajectory of the ensemble. For season onset and peak week, the point forecast is the predicted week of outcome, and for the intensity targets, it is the forecasted intensity rounded to one significant digit. The error in point forecast, 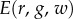, is the absolute error for season onset and season peak week, and the absolute proportional error (error as a proportion of the true outcome) for the remaining targets. 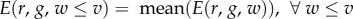 is the average error for target *g* at region *r* for all weeks up to and including week *v*, and 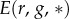 is the average error across all weeks of the season.

#### 2.6.4. Forecast variants

We produced real-time forecasts during the 2016–2017 season and used the scores and errors of these real-time forecasts to evaluate the relative performance of the three methods. In addition to the real-time forecasts, we retrospectively generated the following variant forecasts and calculated their corresponding scores and errors for comparison.

— *Real-time*: *Real-time* forecasts refer to the forecasts produced in real time during the 2016–2017 season as submitted weekly to the CDC influenza forecasting challenge. For these forecasts, small ad hoc changes were made to the three methods throughout the season, sometimes to fix identified software bugs, but more often to improve forecast accuracy based on expert assessment of the ongoing outbreak. For example, given the large outbreaks that occurred in some of the regions, the dynamical models depleted their susceptible populations, which had to be increased to allow for a continued increase in incidence. Similarly, after observing that the distribution of probabilistic forecasts was unrealistically wide, the empirically derived variance of STAT and SE probabilistic forecasts was reduced, based on the evaluation of retrospective forecasts from previous seasons (see the electronic supplementary material).
  In addition to these ad hoc changes, we also made adjustments to the *Real-time* probabilistic forecasts generated from the three approaches, i.e. post-processed the forecasts. This included two adjustments: (i) reduction of the probability assigned by the methods to improbable outcomes (for example, the bins for peak intensity that are lower than the maximal intensity already observed) and (ii) addition of small probabilities to each bin based on historical outbreaks, so as to eliminate the possibility of a 0 probability to the true outcome.
— *Baseline*: The *Baseline* variant of the retrospective forecasts refers to forecasts generated without the ad hoc changes described above; that is, the forecasts for all weeks of the season were generated with the version of the methods current at the end of the season. The resulting scores were compared to the real-time forecasts (*Real-time*) to understand the effect of these changes.
— *Baseline without nowcast*: These forecasts are identical to *Baseline*, except that nowcast information was excluded and real-time CDC ILI alone was used to generate the forecasts. Comparison of scores of this variant with *Baseline* indicates the effect of including nowcast information on forecast accuracy and error.
— *Stable*: CDC ILI estimates for a given week are generally updated for multiple weeks following initial release as some providers submit delayed data (electronic supplementary material, figure S2). The magnitude of these updates varies by region and the period of the season. We considered ILI reported at the end of MMWR week 22 to be final, stable ILI. Retrospective nowcasts and forecasts were generated for MMWR weeks 48 through 18 using this stable ILI. Comparison of scores of this variant with *Baseline* enables the measurement of the effect of revisions to ILI on forecast accuracy and error.
— *Baseline with post-processing*: To assess the effect of the post-processing applied to *Real-time* forecasts (as described above), we also applied the same post-forecast adjustments to the *Baseline* forecasts to create *Baseline with post-processing* forecasts, and accuracy scores were compared to *Baseline* to understand the impact of post-processing. No post-processing was applied to the point forecasts.

An archive of forecasts from the above variants and the calculated evaluation measures are provided as electronic supplementary material.

## 3. Results

### 3.1. Real-time forecasts

**Figure 1.**
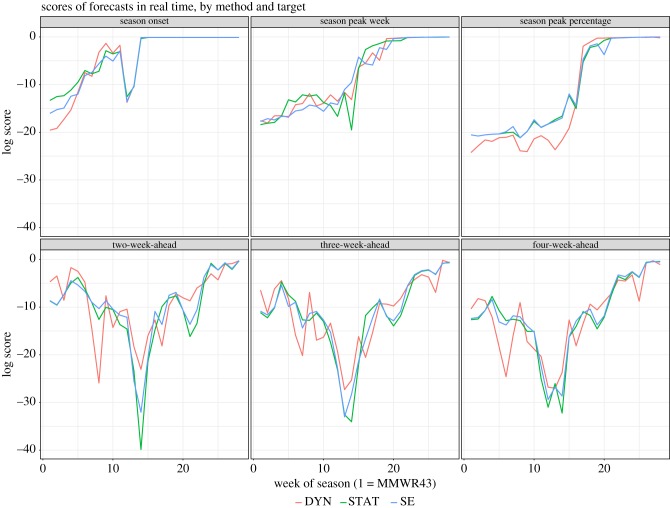
Scores for forecasts at each week of the season, by target. Target ‘one-week-ahead’ was excluded as it would be identical for the three methods.

The cumulative log scores for the *Real-time* probabilistic forecasts through the end of MMWR week 18 are summarized in table 1 and figure 1. Onset occurs relatively early in the season and forecasts of this target in later weeks, after the onset has occurred, are generally correct; consequently, cumulative score for onset was highest among the targets (table 1). All three methods performed better at predicting season peak week than in predicting peak intensity. DYN had better scores for two- and three-week-ahead forecasts than STAT and SE, but consistently underperformed in predicting peak intensity during pre-peak weeks. For the near-term forecasts, for all three methods, lower scores were seen during the weeks of high incidence, i.e. three to four weeks before or after the peak.

**Table 1.** Cumulative log scores and mean errors of the real-time forecast variant for week 48 through week 18 forecasts at the national and 10 HHS regions during the 2016–2017 season. *One-week-ahead* is not displayed as all three methods used nowcasts, and the scores/errors were thus identical. For each target, the best score and lowest error are in italics.

	probabilistic forecasts—log scores			point forecasts—mean errors		
target	DYN	STAT	SE	DYN	STAT	SE
season onset	−134	−*115*	−129	0.884	0.523	*0.516*
season peak week	−*226*	−*226*	−231	1.581	1.604	*1.513*
season peak intensity	−348	−*311*	−*311*	0.165	0.135	*0.129*
two-week-ahead	−*252*	−288	−266	0.204	0.195	*0.193*
three-week-ahead	−*311*	−322	−318	0.251	*0.228*	*0.228*
four-week-ahead	−344	−340	−*329*	0.290	*0.249*	0.254

For the point forecasts, superior performance of SE was more evident. A paired Wilcoxon signed-rank test on point forecast errors (table 2) showed that, for a majority of the targets, DYN had statistically significant larger errors relative to both STAT and SE, but the differences between STAT and SE were not significant (except for peak intensity, for which STAT did significantly better).

**Table 2.** Statistical significance of difference in errors from each forecasting method as determined by a paired Wilcoxon signed-rank test. The values in the parentheses show the *p*-value resulting from testing for alternative hypothesis ‘lesser’ and ‘greater’, respectively. For example, in onset, error with DYN is significantly *greater* (0.01) than error with STAT and error with SE (less than 0.01); and there is no difference in errors of STAT and SE (0.14). For seasonal targets, only weeks prior to the occurrence of the event are used, as forecasts made after the event are almost always correct. See electronic supplementary material, table S1, for significant tests by variant. Statistically significant differences are italicized.

	DYN, STAT	DYN, SE	STAT, SE
season onset	(*0.99, 0.01*)	(*1, 0*)	(0.86, 0.14)
season peak week	(0.65, 0.35)	(0.76, 0.24)	(0.77, 0.23)
season peak intensity	(*1, 0*)	(*1, 0*)	(*1, 0*)
two-week-ahead	(0.84, 0.16)	(*0.99, 0.01*)	(0.8, 0.2)
three-week-ahead	(*0.99, 0.01*)	(*0.99, 0.01*)	(0.83, 0.17)
four-week-ahead	(*1, 0*)	(*1, 0*)	(0.45, 0.55)

**Figure 2.**
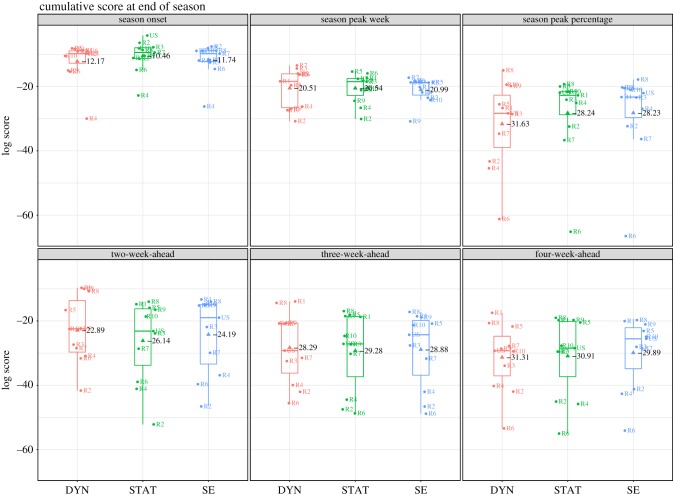
Cumulative score at the end of season by location and target. Target ‘one-week-ahead’ was excluded as it would be identical for the three methods. The boxplot denotes the median, interquartile range (IQR) and the extrema (IQR*1.5). The text in black shows the mean score across the 11 locations.

Figure 2 shows that there is a considerable range of cumulative scores across locations, particularly with the intensity targets. For all three methods, the intensity forecasts for HHS region 6 were among the lowest scoring forecasts possibly due to the large, sustained outbreak observed in this region. Forecasts in regions with smaller outbreaks scored better, but this is quite possibly an artefact of the scoring scheme (as elaborated in the next section).

### 3.2. Effect of real-time adjustment: comparing *Baseline* and *Real-time* forecasts

**Figure 3.**
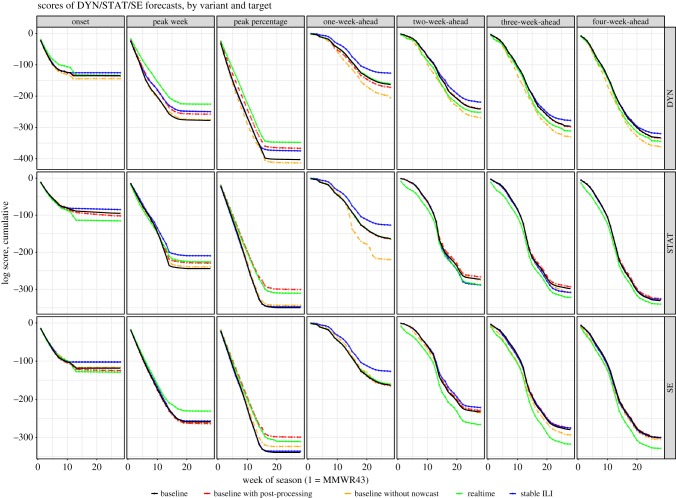
Cumulative sum of log score of the three methods, by variant and target. In each sub-panel, the better scoring variant would have a higher cumulative score, i.e. closer to *y* = 0. For example, with DYN, the one-week-ahead scores for *Baseline*, *Real-time* and *Baseline with post-processing* have very similar scores. Removing nowcast degraded the scores and the availability of stable ILI improved the scores.

We next compare the results of the variant forecasts in table 3 (cumulative probability score) and table 4 (mean point forecast errors). The weekly cumulative log score for each forecast variant, target and forecast method is shown in figure 3. These results show that for all three forecast methods, intra-seasonal real-time adjustments of the *Real-time* forecasts improved the probabilistic forecast scores for the peak week and peak intensity targets, but degraded the near-term forecast scores and, for STAT and SE, the season onset scores. The effect of the adjustments on the mean point forecast errors was less consistent, varying by model and target (table 4). In contrast to the probabilistic forecasts, the DYN point forecasts had small but significant error reductions for near-term targets in the *Real-time* forecasts.

**Table 3.** Cumulative probabilistic forecast scores for all variants. The value in parentheses is the percentage difference relative to the *Baseline* score. Positive numbers in parentheses indicate improved performance and vice versa.

method	target	*Baseline*	*Real-time*	*Baseline without nowcast*	*Baseline with post-processing*	*Stable ILI*
DYN	season onset	−135	−134(1)	−145(−7)	−136(−1)	−125(7)
	season peak week	−278	−226(19)	−276(1)	−258(7)	−250(10)
	season peak intensity	−403	−348(14)	−413(−3)	−367(9)	−375(7)
	one-week-ahead	−163	−161(1)	−205(−25)	−172(−5)	−127(22)
	two-week-ahead	−241	−252(−4)	−269(−12)	−240(0)	−219(9)
	three-week-ahead	−296	−311(−5)	−330(−11)	−298(−1)	−278(6)
	four-week-ahead	−333	−344(−3)	−362(−9)	−335(0)	−320(4)
	*overall*	*−1849*	*−1776*(*4*)	*−1999*(*−8*)	*−1805*(*2*)	*−1693*(*8*)
STAT	season onset	−95	−115(−21)	−94(1)	−102(−7)	−85(11)
	season peak week	−244	−226(7)	−240(2)	−229(6)	−209(14)
	season peak intensity	−350	−311(11)	−343(2)	−301(14)	−347(1)
	one-week-ahead	−163	−163(0)	−220(−35)	−165(−1)	−127(22)
	two-week-ahead	−273	−288(−5)	−275(−1)	−266(2)	−288(−6)
	three-week-ahead	−298	−322(−8)	−308(−3)	−293(2)	−309(−4)
	four-week-ahead	−331	−340(−3)	−326(1)	−325(2)	−327(1)
	*overall*	*−1754*	*−1765*(*−1*)	*−1806*(*−3*)	*−1680*(*4*)	*−1692*(*3*)
SE	season onset	−118	−129(−9)	−116(2)	−125(−5)	−103(13)
	season peak week	−259	−231(11)	−262(−1)	−264(−2)	−257(1)
	season peak intensity	−339	−311(8)	−324(5)	−299(12)	−336(1)
	one-week-ahead	−163	−161(2)	−160(2)	−165(−1)	−127(22)
	two-week-ahead	−233	−266(−14)	−235(−1)	−229(2)	−222(5)
	three-week-ahead	−280	−318(−14)	−293(−5)	−275(2)	−275(2)
	four-week-ahead	−301	−329(−9)	−305(−1)	−300(0)	−300(0)
	*overall*	*−1694*	*−1744*(*−3*)	*−1695*(*0*)	*−1657*(*2*)	*−1619*(*4*)

**Table 4.** Mean point forecast errors for all variants. The value in parentheses is the percentage difference from the *Baseline* error and an italic value indicates that the difference was found to be significant (*p* < 0.05) with a paired Wilcoxon signed-rank test. As no post-processing was applied to the point forecasts, errors with *Baseline with post-processing* are identical to those with *Baseline* and hence omitted.

method	target	*Baseline*	*Real-time*	*Baseline without nowcast*	*Stable ILI*
DYN	season onset	0.784	0.884(−*13*)	0.839(−*7*)	0.709(10)
	season peak week	1.536	1.581(−3)	1.575(−3)	1.5(2)
	season peak intensity	0.169	0.165(3)	0.201(−*19*)	0.17(−1)
	one-week-ahead	0.15	0.147(*2*)	0.185(−*24*)	0.117(*22*)
	two-week-ahead	0.209	0.204(2)	0.269(−*29*)	0.178(*15*)
	three-week-ahead	0.268	0.251(*6*)	0.363(−*35*)	0.257(*4*)
	four-week-ahead	0.327	0.290(*11*)	0.457(−*40*)	0.325(1)
STAT	season onset	0.558	0.523(6)	0.503(10)	0.386(*31*)
	season peak week	1.604	1.604(0)	1.679(−5)	1.627(−1)
	season peak intensity	0.136	0.135(*1*)	0.134(*2*)	0.132(*3*)
	one-week-ahead	0.149	0.147(*2*)	0.148(1)	0.117(*22*)
	two-week-ahead	0.172	0.195(−*13*)	0.182(−6)	0.175(−1)
	three-week-ahead	0.207	0.228(−10)	0.220(−6)	0.21(−2)
	four-week-ahead	0.231	0.249(−8)	0.238(−3)	0.228(1)
SE	season onset	0.546	0.516(5)	0.494(*10*)	0.445(18)
	season peak week	1.442	1.513(−*5*)	1.523(−6)	1.412(2)
	season peak intensity	0.126	0.129(−*3*)	0.123(*2*)	0.122(3)
	one-week-ahead	0.149	0.147(*2*)	0.148(0)	0.117(*22*)
	two-week-ahead	0.165	0.193(−*17*)	0.195(−*18*)	0.161(2)
	three-week-ahead	0.210	0.228(−*8*)	0.257(−*22*)	0.217(−3)
	four-week-ahead	0.243	0.254(−5)	0.284(−*17*)	0.252(−3)

### 3.3. Effect of nowcasts: comparing *Baseline* and *Baseline without nowcasts*

For DYN, the use of nowcast had considerable (8%) benefit overall, especially for the near-term forecasts (table 3 and figure 3). The nowcast also substantially (35%) improved the one-week-ahead forecast for STAT; however, the overall benefit of the nowcast was less pronounced for STAT and SE. Specifically, for the SE method, the use of nowcasts only had a marginal impact on scores and even the one-week-ahead forecasts were found to be comparable with and without nowcasts.

Consistent with the improvement observed for log scores, the DYN point forecasts had significantly lower errors for *Baseline* than *Baseline without nowcast*, especially for the one- to four-week-ahead forecasts (table 4). Fewer significant differences were observed for STAT. For SE, the onset forecasts for *Baseline without nowcast* were better than *Baseline*, and the one-week-ahead forecast was as good as the nowcast (as was also seen for the probabilistic forecasts). However, the two- to four-week-ahead SE forecasts were significantly improved with the use of nowcast.

**Figure 4.**
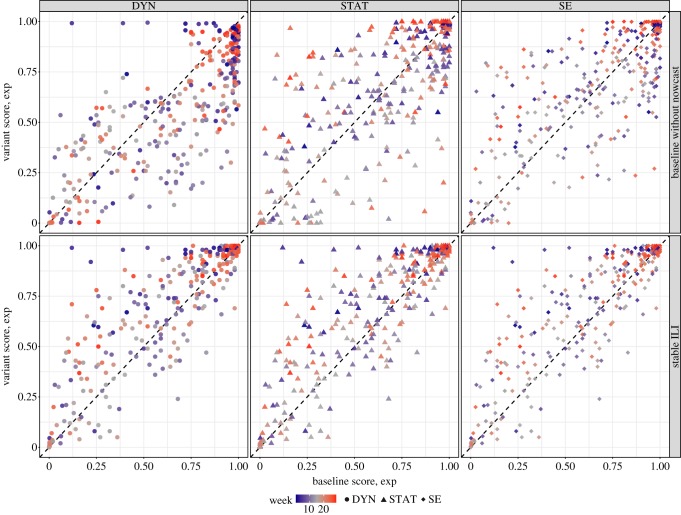
Scores of the probabilistic forecasts for one-week-ahead forecasts from *Baseline* versus one of the variant forms. The colour of the data point denotes the week of the season, and the shape of the data point denotes the forecast method. Points above the diagonal line indicate that the variant (*Baseline without nowcast* for top row; *Stable ILI* for bottom row) outperforms baseline, while points below the diagonal line indicate that *Baseline* results in a higher score. Note that because one-week-ahead forecasts for both the *Baseline* and *Stable ILI* variants are nowcasts, and the same nowcasts are used for DYN, STAT and SE, the three subpanels in the second row are identical. Post-processing does not change nowcast considerably and hence is not shown.

To further compare the performance of the *Baseline without nowcast* variant with the *Baseline* method over the course of the 2016–2017 season, we present, in figure 4, a scatterplot comparing *Baseline* scores (*x*-axis) to the variant scores (*y*-axis) for one-week-ahead forecast. Points above the diagonal line indicate an improvement by the variant method, while dots below indicate a degradation. For *Baseline*, the nowcast is used directly as the one-week-ahead forecast for all three forecast methods. Therefore, the top row (*Baseline without nowcast* versus *Baseline*) compares the accuracy of the nowcast one-week-ahead estimates to that generated by the three forecast methods. This comparison shows that while use of nowcast information improved the DYN forecasts substantially during the very early weeks of the season and during some of the later weeks, such benefits were not seen for SE and STAT.

### 3.4. Effect of post-processing: comparing *Baseline* and *Baseline with post-processing*

Post-processing generally improved cumulative probabilistic forecast scores for all three forecast methods (table 3 and figure 3) and for all targets with the exception of the season onset predictions. The decrease in onset score may have been due to premature elimination of bins based on onset as observed in the moment, which changed in the final revised ILI. The greatest improvements from post-processing were observed for season peak intensity (DYN: 9%; STAT: 14%; SE: 12%).

### 3.5. Effect of transience in CDC ILI estimates: comparing *Stable* to other forecast variants

Forecast accuracy improved for nearly all targets and forecast methods with the use of stable ILI (table 3 and figures 3 and 4). For the nowcasts, these effects were most pronounced during the early and late season when observed ILI levels were lower. The effects of stable ILI on point prediction error were less pronounced (table 4). A statistically significant reduction of nowcast (one-week-ahead forecast) error resulted from the use of stable ILI. For DYN, the unstabilized ILI led to significant increases in error for the two- to three-week-ahead forecasts only. The SE and STAT point forecasts were less sensitive and few other significant differences were found between *Baseline* and *Stable ILI*; the exception was season onset for STAT where the point estimate error decreased with stabilized ILI.

## 4. Discussion

Our analysis of the 2016–2017 forecasts from the DYN, STAT and SE approaches found that SE produced the most accurate *point* forecasts across targets and variants (table 4). The scores of the *probabilistic* forecasts, on the other hand, did not conclusively identify any one approach as optimal. Although SE had the highest *overall* score for all variant sets of forecasts, this was not consistent for all targets and locations. STAT was found to be more accurate in predicting seasonal targets (e.g. seasonal onset), while DYN was found to be better in near-term forecasts.

This discrepancy in SE's advantage over DYN and STAT is likely explained by the fact that the weights applied to individual component models in SE are optimized according to point rather than probabilistic forecast estimates. These results may also indicate a sub-optimal calibration of the SE probability distribution. In particular, for the *Real-time* forecasts, we frequently found the distribution to be unrealistically wide. Furthermore, the SE approach used here assumed a Gaussian probability distribution, whereas STAT and DYN approaches allowed for nonparametric distributions.

These results suggest that, while the multi-model super-ensemble is expected to outperform individual models, there continues to be value in using individual statistical and mechanistic models, and the development and calibration of probabilistic super-ensemble forecasts remains an area of ongoing research.

We see a clear advantage from use of nowcasts, with the size of the effect varying by target and method. This advantage is most pronounced for the one-week-ahead forecast, for which the nowcast provides a more accurate assessment of near-term influenza than that provided by the mechanistic and statistical models. The nowcast additionally improves forecasts of the other targets, as it provides an additional ILI observation beyond what is provided by surveillance data, which is used for training and optimizing the mechanistic and statistical models.

In the idealized experiment assessing the performance of forecast with stable ILI, we found a significant impact of reporting delays on forecast quality. Electronic supplementary material, table S3, demonstrates that this impact of stable ILI is not limited to indirect effect from the improved nowcasts. Given the rather formidable task of gathering data from several thousand physicians, disparate data systems and the need for robust quality checks, reporting lags and revisions in ILINet are expected and understandable. However, our results suggest that a significant improvement in forecast quality could be expected, irrespective of the forecast method, with a reduction in the magnitude of these revisions.

The methods presented here do not incorporate some known characteristics of seasonal influenza outbreaks. For example, these forecasts were generated using ILI which is quite broadly defined and captures illnesses other than influenza. In the past, we have proposed the use of ILI+, a product of ILI and the percentage of virological specimens positive for influenza, as a cleaner signal of influenza. We have also shown that combining separate type-specific (A/H3N2, B etc.) ILI+ forecasts is better than forecasting ILI+, but we have yet to investigate how this circulating type information can be used to improve ILI forecasts. Similarly, while it is known that transmission dynamics are different for children, adults and older adults, and age-stratified ILI information is available through ILINet, we have not attempted to model these sub-populations separately.

The bin sizes and scoring rules presented in this paper are similar to those proposed by FluSight to compare forecasts across participating teams. However, some limitations exist. For example, the fixed interval sizes and the acceptance margins of the intensity targets benefit smaller outbreaks. During the 2016–2017 season, in HHS region 1 and HHS region 8 where the intensities did not exceed 3%, an acceptable margin of ±0.5% makes less of a demand on forecast precision than in regions where the peak intensity was 8–10%. A different scoring scheme where the acceptable margins vary in proportion to outbreak size would weigh outbreaks more equitably and needs to be explored. Similarly, the current scheme weighs forecasts made at each week equally and does not sufficiently account for the higher operational value of the forecasts made during high activity weeks or weeks preceding the peak.

An extension of FluSight real-time forecast to include state-level forecasts has recently been proposed and being implemented for the 2017–2018 season. We believe that these more finely resolved forecasts would be more useful to decision makers than regional forecasts. Mechanistic models, which explicitly consider transmission dynamics in a given population, may be better able to capture infection pathways at the sub-regional scales than at the regional scales. As a consequence, state-level forecasts generated with dynamic models may prove more accurate than regional forecasts, provided that ILI observational estimates are similarly representative of true local infection rates. However, there is no such expectation for the statistical models. It will thus be important to determine whether the differences in accuracy among statistical and mechanistic models at the regional level are reproduced at the state level.

A related extension is an application of these approaches to generate national forecasts for countries where real-time influenza outbreak data are publically available. During the 2017–2018 season, using the model-inference framework described here, we generated and published real-time forecasts of about 35 countries that report ILI data to the World Health Organization [64]. Preliminary results from this season and a retrospective analysis of the forecast quality from up to seven seasons indicate that the model-inference framework can work with data streams other than ILINet used in the USA. In addition, we recently reported an improvement in forecast quality through a networked meta-population forecast system that combined surveillance data and human mobility data to model the spatial movement of influenza in the USA [65]. This system was used operationally in the 2017–2018 FluSight challenge and it would be interesting to further evaluate its utility at supranational scales. Overall, our results suggest that:

— The BMA super-ensemble has better overall accuracy but does not conclusively outperform the individual models, and exploration of modifications and/or alternatives is required.
— Transient errors in surveillance data considerably degrade the accuracy of the forecasts.
— Reliable non-surveillance proxies of influenza incidence, when available and appropriately used, could improve forecasts and partially address reporting delays.
— The methods need to be more robust and less dependent on ad hoc or post hoc manual changes.

## Supplementary Material

Additional descriptions of methods, supporting tables and plots

## Supplementary Material

Data - Climatological specific humidity for HHS regions

## Supplementary Material

Data- Evaluation measures for the forecasts

## Supplementary Material

Data - Forecast archive

## Supplementary Material

Code - R script to gnerate manuscript plots and table

## Endnotes

1 This differs from the FluSight guidelines where onset is defined as the first of three consecutive weeks with observed ILI greater than *or equal to* the region-specific baseline.

2 When none of the true outcomes have a non-zero probability assigned to them, as ln(0) is −∞, the score is assumed to be −10. This is consistent with the 2016–2017 FluSight guidelines.

## Data accessibility

Datasets of the forecasts, evaluation measures and code to reproduce the tables and plots of this paper are provided as electronic supplementary material.
