# Supplementary material for: Evaluation of mechanistic and statistical methods in forecasting influenza-like illness
Source: J R Soc Interface. 2018 Jul 25;15(144):20180174. doi: 10.1098/rsif.2018.0174 (PMC6073642; doi:10.1098/rsif.2018.0174)
Supplement: Additional descriptions of methods, supporting tables and plots [file rsif20180174supp1.docx]

### **Evaluation of Mechanistic and Statistical Methods in Forecasting Influenza-like Illness**

Sasikiran Kandula, Teresa Yamana, Sen Pei, Wan Yang, Haruka Morita, Jeffrey Shaman

Department of Environmental Health Sciences, Columbia University, New York, NY.

### **Supporting Information**

Corresponding author:

Sasikiran Kandula

Dept. of Environmental Health Sciences,

722 West 168^th^ Street, 11^th^ Floor

Columbia University, New York, NY. 10032

[sk3542@cumc.columbia.edu](mailto:sk3542@cumc.columbia.edu)

**Data assimilation methods**

*The Ensemble Adjustment Kalman Filter with error correction (EAKFC)*

The EAKFC consists of an EAKF step for training the model and an error correction step following the last EAKF update, as described in turn below. The Ensemble Adjustment Kalman Filter (EAKF) uses a model ensemble to approximate the distribution of the modeled system. For the observed variable, the EAKF updates the posterior of each ensemble member sequentially each week, based on the prior and the observation per the following equation:

$x_{t,post}^{n}=\left( \frac{{OEV}_{t}}{{OEV}_{t}+\sigma_{t,prior}^{2}}\bar{x_{t,prior}}+\frac{\sigma_{t,prior}^{2}}{{OEV}_{t}+\sigma_{t,prior}^{2}}z_{t} \right)+\sqrt{\frac{{OEV}_{t}}{{OEV}_{t}+\sigma_{t,prior}^{2}}}(x_{t,prior}^{n}-\bar{x_{t,prior}})$ [S1]

where $x_{t, post}^{n}$ is the posterior estimate of the observed variable (modeled incidence here) at week *t*, for the *n*th ensemble member. $x_{t,prior}^{n}$ is the corresponding prior estimate (i.e. the 1-step ahead prediction per the SEIRS model described in the main text). $\bar{x_{t,prior}}$and $\sigma_{t, prior}^{2}$ are the prior ensemble mean and variance, respectively, and are both computed directly from the ensemble. The observation, $z_{t}$, was taken as the observed influenza-like illness (ILI) record multiplied by an arbitrary factor (0.75 here based on testing of historical data) (1, 2). ${OEV}_{t}$ is the observational error variance (OEV) at time *t*; as in our previous work (1), here we adopted a heuristic estimate per ${OEV}_{t}=1\times{10}^{5}+ {(\sum_{j=t-3}^{t-1} z_{j}/3)}^{2}/5$, which contains a baseline uncertainty (1^st^ summand) and a proportional part determined by observations during the preceding 3 weeks (2^nd^ summand). Note that, on the RHS of Eqn. S1, the entire term in the first parentheses is the *posterior* ensemble mean and the 2^nd^ summand is the adjustment to each ensemble member. For each unobserved state (variable/parameter), the EAKF updates its posterior based on its collinearity with the observed variable. Specifically, the posterior increment of the unobserved state is computed as the product of the posterior increment of the observed variable (i.e. ${x_{t, post}^{n}-x}_{t, prior}^{n}$) and the ratio of the state-observed variable covariance to the prior variance of the observed variable (i.e. $\sigma_{t, prior}^{2}$).

Although the EAKF is optimal for linear models, for nonlinear models such as the SEIRS model used here, the assumption of linear covariability is not strictly satisfied. Consequently, errors in sensitive parameters/variables can grow in a nonlinear way. In our previous work, we found that this nonlinear error growth tends to follow a defined structure and that, by counteracting these structural errors, the forecast accuracy of EAKF can be improved (3). In our implementation, we focus on two of the more sensitive system states – the susceptible population S and the maximal basic reproductive number $R_{0max}$. Briefly, in the dynamical error corrected EAKF(3), we first diagnose the nonlinear error structure between S, $R_{0max}$ and weekly incidence using error breeding (4, 5). The manifold of error structure is then fitted with a 3rd-order polynomial, from which the errors in S and $R_{0max}$ are inferred from the discrepancy between observed variable with observations. These sensitive state variables are then additionally updated by counteracting the diagnosed structural errors, while the adjustment of other state variables and parameters remains unchanged.

To avoid inappropriate error corrections that might undermine accurate EAKF update, the error correction procedure is only applied when clear error structure emerges, which usually occurs after the epidemic onset (3). Based on retrospective forecasts of outbreaks from 1997/98 through 2015/16 seasons, we found that the greatest improvement in forecast quality occurs when the error correction is applied only after the cumulative incidence in a season exceeds one third of the typical annual incidence averaged over all historical outbreaks at the location (Figure S3).

*Ensemble Kalman Filter (EnKF)*

The EnKF in this study (6) is a stochastic ensemble Kalman filter variant. The main difference between the EAKF and the EnKF is that, for the EnKF random noise is added to the observation. Specifically, the EnKF adjusts the posterior observed variable per the following equation:

$x_{t,post}^{n}=\frac{{OEV}_{t}}{{OEV}_{t}+\sigma_{t,prior}^{2}}x_{t,prior}+\frac{\sigma_{t,prior}^{2}}{{OEV}_{t}+\sigma_{t,prior}^{2}}\tilde{z_{t}}$ [S2]

where $\tilde{z_{t}}$ is the perturbed observation with a Gaussian noise randomly drawn from Norm(0, ${OEV}_{t}$). Other settings are the same as the EAKF. No error correction was applied to the EnKF.

*Rank Histogram Filter (RHF)*

Similar to the EAKF and EnKF, the RHF (7) also uses a model ensemble to represent the distribution of the model system. However, unlike the former two filters, the RHF does not assume linearity of the system and thus the observation likelihood can assume any form of distribution (as opposed to the implicit Gaussian distribution for Kalman filters). For the observed variable, the RHF computes the posterior directly following Bayes’ theorem, i.e. by taking the product of the prior and the likelihood. As in the EAKF and EnKF, the prior is computed based on the dynamic model (i.e. 1-step ahead prediction per the SEIRS model in this study). The likelihood here, which can be any nonlinear distribution, was taken as a Gaussian as in the EAKF, i.e. Norm(*z_t_*, ${OEV}_{t}^{2}$). The ensemble is first ranked (hence the name) and the *n*th ensemble member (i.e. the *n*th largest among the ensemble) is updated such that the cumulative posterior density at that updated ensemble member equals *n*/(*N*+1) with *N* being the ensemble size (*N*=300 here). For the unobserved state, the posterior is computed in the same way as in the EAKF.

**Probabilistic and Point forecasts**

*DYN*

Each ensemble of the SEIRS-EAKFC model, has 300 ensemble members all of which are initialized with random draws from the same parameter ranges and fit independently of each other. Thus we have posteriors and forecasts from each ensemble member. For each target and possible outcome, the probability assigned to a possible outcome is the proportion of ensemble members predicting the outcome. To get point predictions, we calculated the mean trajectory of the 300 ensemble members i.e. the ILI at week *t* of the mean trajectory is the mean of the 300 estimates of ILI at *t*. The point estimates of the seven targets are computed from the mean trajectory.

### STAT

STAT forecasts of season onset and season peak week used an equal-weighted average of the BWO and KNN forecasts for both point estimates and probabilistic forecasts.

Point estimate forecasts for season peak intensity and incidence one- to four-weeks ahead were produced using a weighted average of the BWO and KNN forecast point estimates. These weights were calculated using Bayesian model averaging method (described for the SE forecast below), and are based on the performance of the forecasts produced by these two statistical methods during the training period spanning the 2004/05 through 2015/16 influenza seasons, excluding the pandemic years of 2008/09 and 2009/10.

Probabilistic forecasts of season peak intensity and one- to four-week ahead forecasts were assumed to be normally distributed with mean equal to the weighted-average point estimate, and a variance, specific to each week and forecast target, assigned based on empirical tests of retrospective forecasts. For season peak intensity, the variance was computed as in (8), with a component representing variance between the individual point estimates (between-forecast variance) and a component representing the variance around each individual forecast (within-forecast variance). For simplicity, individual forecasts are assumed to have identical variance, which is determined by the Bayesian model averaging algorithm. The one- to four-week ahead forecasts used the historically observed variance of ILI(*t+w*)-ILI(*t*), where *t* is the week of forecast, and *w* is one through four weeks ahead.

### SE

SE point estimates for all targets were calculated using Bayesian model averaging to produce weighted averages of the point estimates from eight individual models: the two statistical models (BWO and KNN), and six model-filter systems (EAKFC, EnKF or RHF data-assimilation methods combined with either an SIRS or SEIRS model structure).

The weights assigned to each component forecast point-estimate are determined using maximum likelihood estimation of the following, over the training period:

|  | $p\left( y \vert F_{1},\ldots,F_{8} \right)=\sum_{k=1}^{8} w_{k}g_{k}(y\vert F_{k})$ | [S3] |
| --- | --- | --- |

where *y* is the forecast target, *w_k_* is the probability that forecast method *k* is the most accurate method, and *g_k_(y|F_K_)* is the probability distribution function (PDF) of *y*, conditional on *F_k_,* given that *F_k_* is the most accurate forecast. This conditional PDF is assumed to be normal with mean *F_k_* and standard deviation σ. For simplicity, we assume equal σ for all component forecasts (see (8) for full details).

As in STAT, the training period spans the 2004/05 through 2015/16 influenza seasons, excluding the pandemic years of 2008/09 and 2009/10. Although ILI from 1997/98 season are available, as our nowcasts use GET that is only available from 2004, we exclude seasons prior to 2004/05. Weights were computed separately for each target, and for each week.

SE probabilistic forecasts were constructed using normal distributions with mean equal to the weighted-average point estimate forecast, and a variance, specific to each week and forecast target. For seasonal targets (season onset, peak week and peak intensity) the variance was computed as the sum of the variance between the individual point estimates (between-forecast variance) and the component representing the variance around each individual forecast (within-forecast variance), σ^2^, which is estimated by the BMA algorithm in equation S3 (see (8)).

The one- to four-week ahead forecasts use the historically observed variance of ILI(*t+w*)-ILI(*t*), where *t* is the week of forecast, and *w* is one through four weeks ahead.

In the *Real-time* forecasts, it was observed that the probabilistic forecasts were unrealistically wide compared to historically observed variability. Several alternate formulations for forecast variance were tested using empirical tests of retrospective forecasts. We decided to apply an ad-hoc adjustment to the variance of SE National forecasts beginning MMWR Week 3 of 2017, whereby the σ^2^ estimated in S3 was divided by 2, resulting in narrower probability distributions. The variance of 1- through 4- week ahead forecasts for both SE and STAT was further decreased beginning MMWR Week 12. In this case, σ^2^ at week *t* was replaced by the average of σ^2^ estimated for week *t* and the smaller σ^2^ estimated for the final week of the previous season (MMWR Week 18).

See Figure S1 for an example plot of probabilistic forecasts.

**Table S1**. Statistical significance of difference in errors from each forecasting method as determined by a paired Wilcoxon signed rank test. The values in the parenthesis show the P value resulting from testing for alternative hypothesis 'lesser' and 'greater' respectively. For example, DYN's 1-week ahead *Baseline* variant errors, are lesser than *Baseline w/o Nowcast* errors, but greater than errors of *Stable ILI* and of *Real-time.* For seasonal targets only weeks prior to the occurrence of the event are used, as forecasts made after the event are almost always correct.

| **Method** | **Target** | ***Baseline,***  ***Baseline without Nowcast*** | ***Baseline, Stable ILI*** | ***Baseline, Realtime*** | ***Baseline without Nowcast, Stable ILI*** | ***Baseline without Nowcast, Realtime*** | ***Stable ILI, Realtime*** |
| --- | --- | --- | --- | --- | --- | --- | --- |
| **DYN** | Season onset | **(.05, .95)** | (.26, .74) | **(.05, .95)** | **(.95, .05)** | (.45, .55) | (.23, .77) |
|  | Season peak week | (.55, .45) | (.74, .26) | (.06, .94) | (.71, .29) | (.07, .93) | **(.05, .95)** |
|  | Season peak percentage | **(0, 1)** | (.08, .92) | (.07, .93) | **(.96, .04)** | (.53, .47) | (.15, .85) |
|  | 1-week ahead | **(0, 1)** | **(1, 0)** | **(.99, .01)** | **(1, 0)** | **(1, 0)** | **(0, 1)** |
|  | 2-week ahead | **(0, 1)** | **(1, 0)** | (.87, .13) | **(1, 0)** | **(1, 0)** | **(0, 1)** |
|  | 3-week ahead | **(0, 1)** | **(.98, .02)** | **(.99, .01)** | **(1, 0)** | **(1, 0)** | (.43, .57) |
|  | 4-week ahead | **(0, 1)** | (.83, .17) | **(1, 0)** | **(1, 0)** | **(1, 0)** | **(.98, .02)** |
| **STAT** | Season onset | (.94, .06) | **(.98, .02)** | (.87, .13) | (.79, .21) | (.22, .78) | (.08, .92) |
|  | Season peak week | (.65,.35) | (.7, .3) | (.93, .07) | (.45, .55) | (.78, .22) | (.83, .17) |
|  | Season peak percentage | **(.99, .01)** | **(.03, .97)** | **(.99, .01)** | **(0, 1)** | (.31, .69) | **(1, 0)** |
|  | 1-week ahead | (.61, .39) | **(1, 0)** | **(.97, .03)** | **(1, 0)** | (.52, .48) | **(0, 1)** |
|  | 2-week ahead | (.13, .87) | (.47, .53) | **(.03, .97)** | (.68, .32) | (.34, .66) | **(.05, .95)** |
|  | 3-week ahead | (.33, .67) | (.57, .43) | (.09, .91) | (.85, .15) | (.72, .28) | (.12, .88) |
|  | 4-week ahead | (.53, .47) | (.88, .12) | (.41, .59) | (.74, .26) | (.70, .30) | (.06, .94) |
| **SE** | Season onset | **(.97, .03)** | (.29, .81) | (.80, .20) | **(.05, .95)** | (.30, .70) | (.85, .15) |
|  | Season peak week | (.38, .62) | (.77, .23) | **(.03, .97)** | (.82, .18) | (.16, .84) | (.06, .94) |
|  | Season peak percentage | **(1, 0)** | (.07, .93) | **(.05, .95)** | **(0, 1)** | **(0, 1)** | (.51, .49) |
|  | 1-week ahead | (.88, .12) | **(1, 0)** | **(.98, .02)** | **(1, 0)** | (.22, .78) | **(0, 1)** |
|  | 2-week ahead | **(0, 1)** | (.87, .13) | **(0, 1)** | **(1, 0)** | (.65, .35) | **(0, 1)** |
|  | 3-week ahead | **(0, 1)** | (.31, .69) | **(0, 1)** | **(1, 0)** | (.94, .06) | **(.04, .96)** |
|  | 4-week ahead | **(0, 1)** | (.48, .52) | (.19, .81) | **(1, 0)** | **(.95, .05)** | (.17, .83) |

**Table S2**. Based on a preliminary retrospective analysis of forecasts from the past seasons, in addition to the three real-time forecasts described here, we submitted to FluSight in 2016/17 another version that was a combination of forecasts selected from the three methods based on target-specific performance. This selection was not adaptive and remained the same over the season. The forecast method selected for each target was as follows: SIRS-EAKF for onset, STAT for peak week, SE for peak intensity, nowcast for 1-week ahead, and SIRS-EAKFC for 2- to 4-week ahead. Scores from this method were found to be similar to those of SE and only errors in 2-, to 4-week were significantly larger. Cumulative log scores and mean errors for the target specific selection method are shown below. *1-week ahead* is not displayed as this is identical to the other three methods described in the manuscript. An asterisk next to SEL error indicates that the difference (p < .05) relative to SE errors was found to be statistically significant with a Wilcoxon signed rank test.

| **Targets** | **Prob. forecasts – Log score** | | **Point forecasts - Errors** | |
| --- | --- | --- | --- | --- |
|  | **SEL** | **SE** | **SEL** | **SE** |
| Season onset | -118 | -129 | 0.693 | 0.516 |
| Season peak week | -226 | -231 | 1.604 | 1.513 |
| Season peak intensity | -311 | -311 | 0.129 | 0.129 |
| 2-week ahead | -256 | -266 | 0.210* | 0.193 |
| 3-week ahead | -316 | -318 | 0.261* | 0.228 |
| 4-week ahead | -347 | -329 | 0.297* | 0.254 |

**Table S3**. Cumulative probabilistic forecast scores and mean point forecast errors for the variant that forecasts with stable ILI but does not use nowcasts (*Stable ILI without nowcast****)***. Scores/errors for the other two variants are as in Table 3. The value in parenthesis is the percentage difference relative to *Baseline* score. Statistically significant differences in errors relative to *Baseline,* as estimated with a Wilcoxon signed rank test (p < .05), are in bold. A comparison of columns *Baseline without nowcast* and *Stable ILI without nowcast* better isolates the effect of transience on forecast quality.

| **Method** | **Target** | ***Probabilistic Forecasts – Log score*** | | | ***Point Forecasts – Mean errors*** | | |
| --- | --- | --- | --- | --- | --- | --- | --- |
|  |  | ***Baseline without nowcast*** | ***Stable ILI*** | ***Stable ILI without nowcast*** | ***Baseline without nowcast*** | ***Stable ILI*** | ***Stable ILI without nowcast*** |
| **DYN** | Season onset | -145(-7) | -125 (7) | -136(-1) | 0.839**(-7)** | 0.709(10) | 0.747(5) |
|  | Season peak week | -276(1) | -250 (10) | -268(4) | 1.575(-3) | 1.5(2) | 1.520(1) |
|  | Season peak intensity | -413(-3) | -375 (7) | -381(5) | 0.201**(-19)** | 0.17(-1) | 0.184(-9) |
|  | 1-week ahead | -205(-25) | -127 (22) | -184(-13) | 0.185**(-24)** | 0.117**(22)** | 0.159(-6) |
|  | 2-week ahead | -269(-12) | -219 (9) | -252(-5) | 0.269**(-29)** | 0.178**(15)** | 0.233(-12) |
|  | 3-week ahead | -330(-11) | -278 (6) | -313(-5) | 0.363**(-35)** | 0.257**(4)** | 0.329(-23) |
|  | 4-week ahead | -362(-9) | -320 (4) | -345(-3) | 0.457**(-40)** | 0.325(1) | 0.422(-29) |
|  | *Overall* | *-1999(-8)* | *-1693 (8)* | *-1878*(-2) |  |  |  |
| **STAT** | Season onset | -94(1) | -85(11) | -85(10) | 0.503(10) | 0.386**(31)** | 0.386**(31)** |
|  | Season peak week | -240(2) | -209(14) | -258(-6) | 1.679(-5) | 1.627(-1) | 1.714(-7) |
|  | Season peak intensity | -343(2) | -347(1) | -336(4) | 0.134**(2)** | 0.132**(3)** | 0.125(9) |
|  | 1-week ahead | -220(-35) | -127(22) | -196(-20) | 0.148(1) | 0.117**(22)** | 0.134**(10)** |
|  | 2-week ahead | -275(-1) | -288(-6) | -261(4) | 0.182(-6) | 0.175(-1) | 0.170(1) |
|  | 3-week ahead | -308(-3) | -309(-4) | -305(-2) | 0.220(-6) | 0.21(-2) | 0.222(-7) |
|  | 4-week ahead | -326(1) | -327(1) | -324(2) | 0.238(-3) | 0.228(1) | 0.235(-2) |
|  | *Overall* | *-1806(-3)* | *-1692(3)* | *-1764*(-1) |  |  |  |
| **SE** | Season onset | -116(2) | -103(13) | -105(11) | 0.494**(10)** | 0.445(18) | 0.416(24) |
|  | Season peak week | -262(-1) | -257(1) | -265(-2) | 1.523(-6) | 1.412(2) | 1.490(-3) |
|  | Season peak intensity | -324(5) | -336(1) | -316(7) | 0.123(**2**) | 0.122(3) | 0.112(11) |
|  | 1-week ahead | -160(2) | -127(22) | -136(17) | 0.148(0, 1) | 0.117**(22)** | 0.132**(11)** |
|  | 2-week ahead | -235(-1) | -222(5) | -216(7) | 0.195**(-18)** | 0.161(2) | 0.176(-7) |
|  | 3-week ahead | -293(-5) | -275(2) | -278(1) | 0.257**(-22)** | 0.217(-3) | 0.250(-19) |
|  | 4-week ahead | -305(-1) | -300(0) | -298(1) | 0.284**(-17)** | 0.252(-3) | 0.278(-15) |
|  | *Overall* | *-1695(0)* | *-1619(4)* | *-1614(5)* |  |  |  |

**Figure S1**. Probabilistic forecasts at US National for all targets. The week of submission is on the x-axis and valid intervals on the y-axis. Color (low = yellow, high = red) denotes the likelihood that the true outcome will be in a specific bin. The black lines/points show the true outcome value. Note that after the week of true outcome, the distributions of season onset, peak week and peak percentage are quite narrow and are centered on the true outcome.


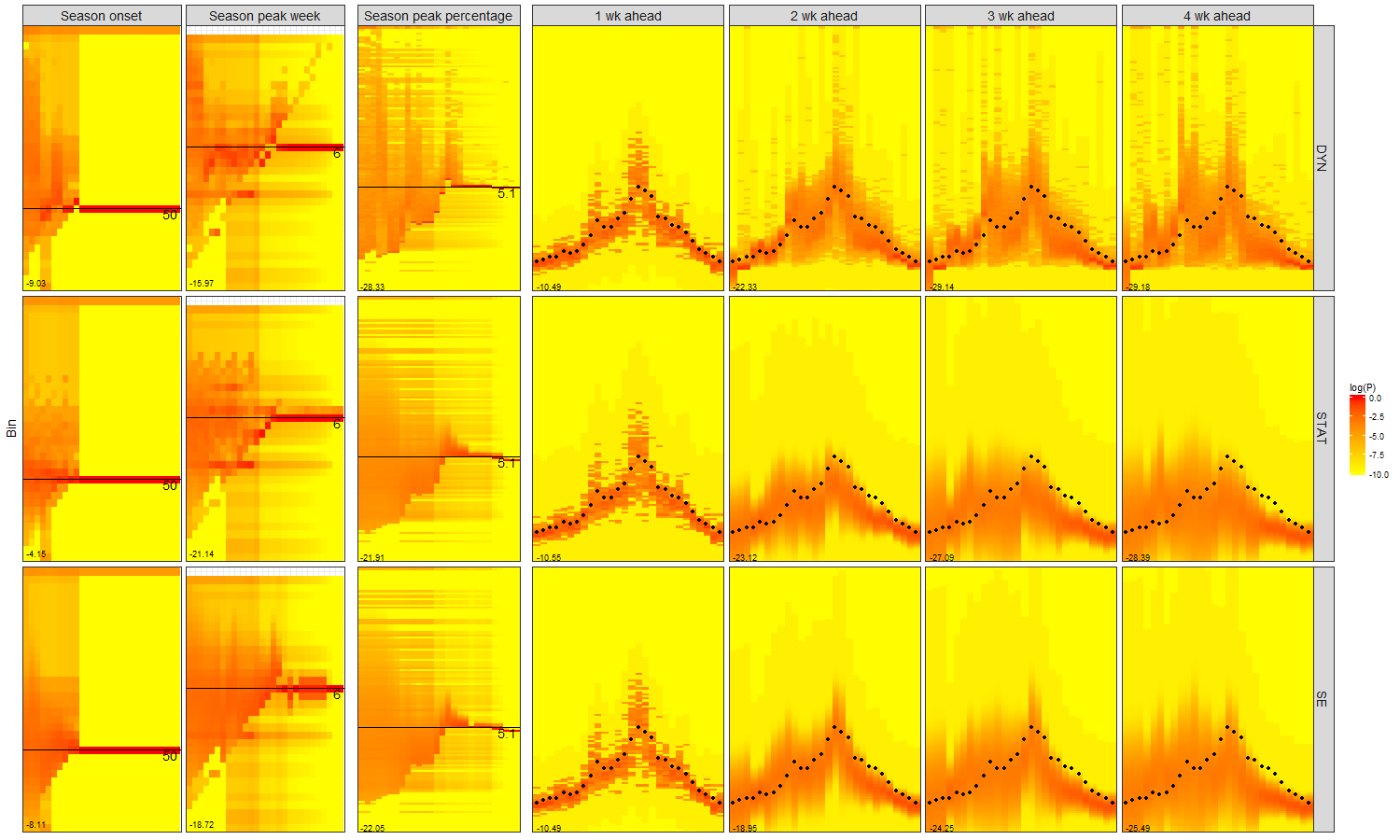


**Figure S2**. ILI and revisions to ILI over time by location. HHS region 6 had very few updates to the ILI in contrast to HHS Region 9 which had large initial under-reporting throughout the season. Over-estimates in Regions 1 and 3 around the peak week are of concern as they can lead to large errors, particularly in peak intensity.


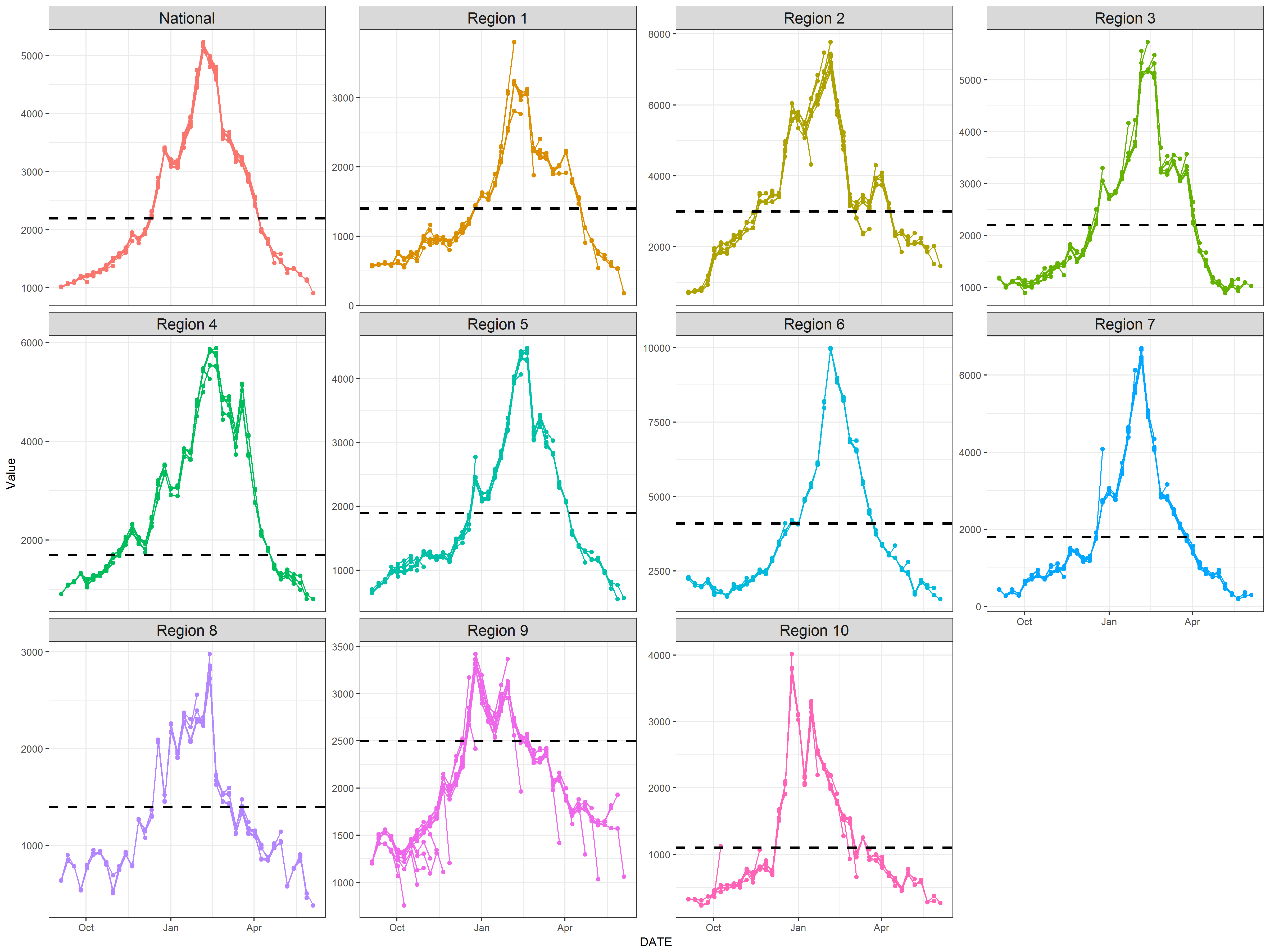


**Figure S3**. For different implementation settings, we compare the average scores of all 7 targets in all regions (national and 10 HHS regions). Two different algorithms – EAKF and EAKF with error correction (EAKFC) – with different scaling parameters (0.75 versus 1) and OEVs (large OEV ${OEV}_{t}=1\times{10}^{5}+ {(\sum_{j=t-3}^{t-1} {ILI}_{j}/3)}^{2}/5$ versus small OEV ${OEV}_{t}=1\times{10}^{4}+ {(\sum_{j=t-3}^{t-1} {ILI}_{j}/3)}^{2}/20$) are compared. For EAKFC, we tested different choices of adaptive threshold values ranging from 0 to 1. Results indicate that the choice of around 33% can achieve the best performance, regardless of the choice of scaling parameter and OEV.


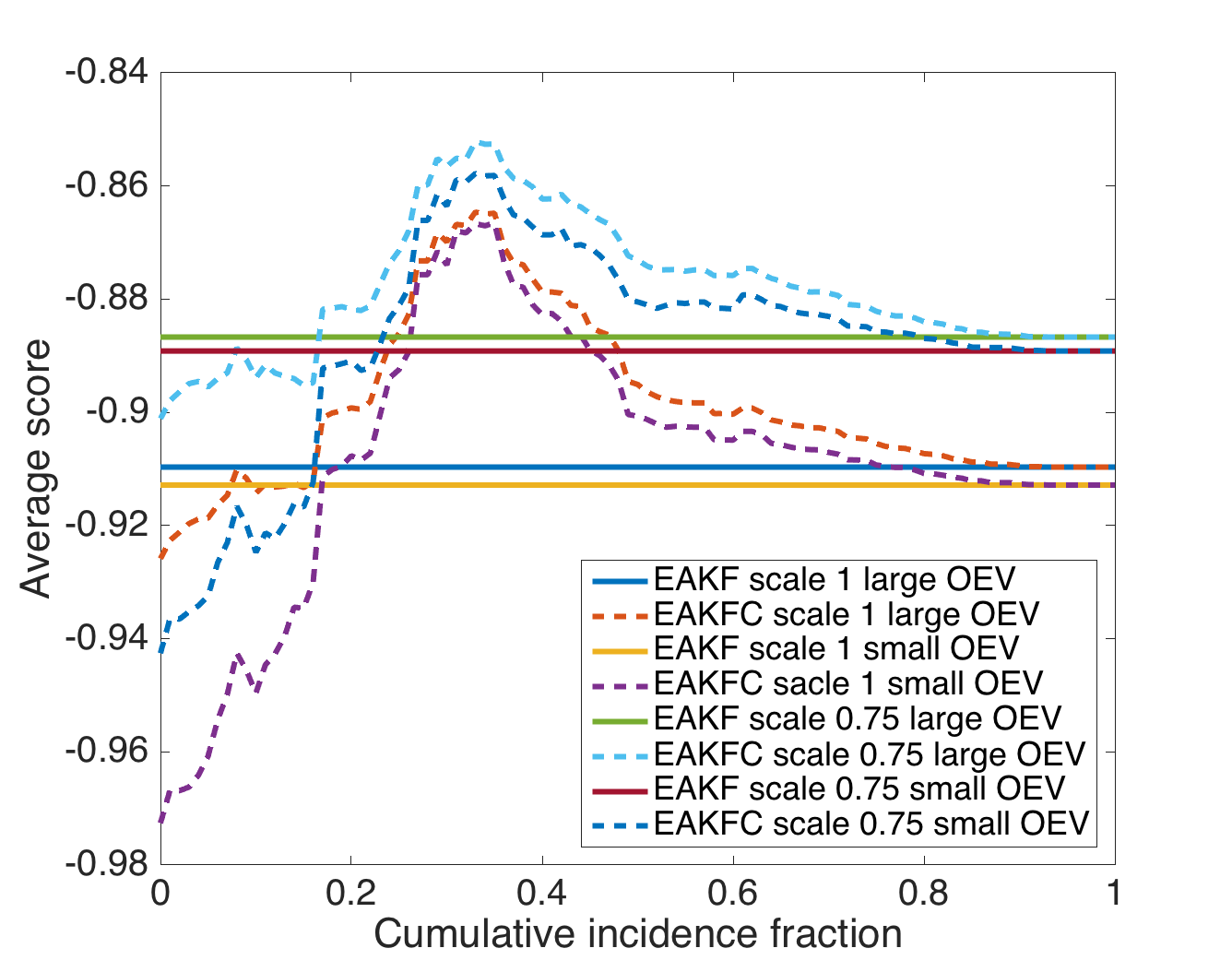


**References**

1. Shaman J, Karspeck A. Forecasting seasonal outbreaks of influenza. Proceedings of the National Academy of Sciences. 2012;109(50):20425-30.

2. Shaman J, Karspeck A, Yang W, Tamerius J, Lipsitch M. Real-time influenza forecasts during the 2012–2013 season. Nature communications. 2013;4:2837.

3. Pei S, Shaman J. Counteracting structural errors in ensemble forecast of influenza outbreaks. Nature communications. 2017;8(1):925.

4. Toth Z, Kalnay E. Ensemble forecasting at NMC: The generation of perturbations. Bulletin of the american meteorological society. 1993;74(12):2317-30.

5. Toth Z, Kalnay E. Ensemble forecasting at NCEP and the breeding method. Monthly Weather Review. 1997;125(12):3297-319.

6. Evensen G. Data assimilation: the ensemble Kalman filter: Springer Science & Business Media; 2009.

7. Anderson JL. A non-Gaussian ensemble filter update for data assimilation. Monthly Weather Review. 2010;138(11):4186-98.

8. Yamana TK, Kandula S, Shaman J. Individual versus superensemble forecasts of seasonal influenza outbreaks in the United States. PLoS computational biology. 2017;13(11):e1005801.
